# Immunoinflammatory Response in Critically Ill Patients: Severe Sepsis and/or Trauma

**DOI:** 10.1155/2013/362793

**Published:** 2013-11-24

**Authors:** Maja Surbatovic, Milic Veljovic, Jasna Jevdjic, Nada Popovic, Dragan Djordjevic, Sonja Radakovic

**Affiliations:** ^1^Clinic of Anesthesiology and Intensive Therapy, Military Medical Academy, Crnotravska 17, 11000 Belgrade, Serbia; ^2^Faculty of Medicine of the Military Medical Academy, University of Defence, Crnotravska 17, 11000 Belgrade, Serbia; ^3^Clinical Center Kragujevac, Zmaj Jovina 30, 34000 Kragujevac, Serbia; ^4^Faculty of Medical Sciences, University of Kragujevac, Svetozara Markovica 69, 34000 Kragujevac, Serbia; ^5^Institute for Infectious and Tropical Diseases, Intensive Care Unit, Clinical Center of Serbia, Pasterova 2, 11000 Belgrade, Serbia; ^6^School of Medicine, University of Belgrade, Dr. Subotica 8, 11000 Belgrade, Serbia; ^7^Sector of Preventive Medicine, Military Medical Academy, Crnotravska 17, 11000 Belgrade, Serbia

## Abstract

Immunoinflammatory response in critically ill patients is very complex. This review explores some of the new elements of immunoinflammatory response in severe sepsis, tumor necrosis factor-alpha in severe acute pancreatitis as a clinical example of immune response in sepsis, immune response in severe trauma with or without secondary sepsis, and genetic aspects of host immuno-inflammatory response to various insults in critically ill patients.

## 1. Some of the New Elements of Immunoinflammatory Response in Severe Sepsis

Infection has been the leading cause of death in humans, since the earliest written sources. In the early 15th century, the black death plague wiped out between one-third and one-half of the entire European and Asian populations. The Greek word “sepo,” from which the term “sepsis” derives, means “decomposition of animal, or vegetable or organic matter in the presence of bacteria” [1]. Modern cytokine research began in 1932, with the pioneer work of Rich and Lewis, who first observed antigen-mediated inhibition of leukocyte migration in tuberculin-sensitized tissue. Cytokine biology expanded 30 years ago, and the term “cytokine” was first used by Cohen, referring to the variety of soluble factors, with wide range of biological activities related to immune system, produced by wide range of cell types [2]. In 1975, Carswell described the pivotal role of tumor necrosis factor, as one of the earliest monokines, in severe sepsis [3]. At first, this cytokine was also called “cahectin,” which describes its ability to suppress lipoprotein lipase activity, leading to hypertriglyceridemia and rapid weight loss in experimental animals [4]. Interactions between infecting microorganisms and host response can lead to severe sepsis and septic shock. In response to pathogen adherence to an epithelial surface, the host initiates specific mucosal defense mechanisms, in order to prevent microbial invasion. The critical bacterial density needed to initiate an infection is called quorum. Bacterial cell-to-cell communication enables them to assess their population density and interact with the host as a population (quorum-sensing systems). Innate immunity—representing early non-specific response system—and adaptive immunity—representing more pathogen-specific response system—are parts of immune system as a whole [5]. The inflammatory response of the host is similar, regardless of the nature of the stimuli (infectious or noninfectious, like tissue injury). Initially, microorganisms bind to surface Toll-like receptors (TLRs) on phagocytic cells. These receptors are homologues of the *Drosophila* Toll protein. This binding initiates a series of intracellular events resulting in the release of cytokines. TLR-2 type of these receptors reacts with Gram-positive bacterial cell wall antigens, such as peptidoglycans and lipoteichoic acid, while TLR-4 form reacts with lipopolysaccharide (LPS) and endotoxin of Gram-negative bacteria [6–8]. TLRs may recognize either pathogens or endogenous danger signals released by stressed or damaged cell and consequently alert the host by activating the innate immune system. Some molecular fragments from pathogens, such as LPS and bacterial DNA, may induce an immune response and are known as specific patterns called pathogen associated molecular patterns (PAMPs). These patterns are recognized by cellular receptors termed pattern recognition receptors (PRRs). Besides PAMPs, there are also several endogenous molecules, such as high-mobility group box- (HMGB-) 1, hyaluronan, and heat-shock proteins (HSPs) that are also able to trigger the immune response through PRRs. These signals are normal cell constituents, which may be released either passively (by necrotic cells) or actively (by stressed cell, in response to cellular injury). Endogenous analogues of PAMPs are called alarmins. These endogenous alarmins and exogenous PAMPs represent two subgroups of the larger category of danger signals termed damage associated molecular patterns (DAMPs) [9]. The explanation of SIRS in absence of obvious microbial infection was provided by Matzinger [10], thus elucidating host response to DAMPs that can activate innate immunity through, among others, TLRs.

Severe sepsis and/or trauma complicated with multiple organ dysfunction syndrome (MODS) are leading causes of death in intensive therapy units with mortality rate exceeding 50%. Besides the infection, the intensity of immunoinflammatory response also influences the outcome because it is essential for host defense. Unfortunately, if this reaction is uncontrolled, it can lead to the MODS [11]. Resident macrophages and polymorphonuclear cells (PMCs) initiate the primary host response to the invading microorganisms, for they are responsible for the primary phagocytosis and subsequent activation and recruitment of polymorphonuclear granulocytes and monocytes. The macrophage population increase by rapid differentiation of monocytes. This concerted action which constitutes the innate response to infections and tissue damage is mediated by various soluble and membrane-bound factors. Cytokines are potent, low molecular weight proteins produced by nucleated cells, particularly those of the immune system, which exert control over the duration and amplitude of the immune/inflammatory response. They are the main positive and negative regulators of immune responses as well as the key components in the integration of these reactions with other physiological systems such as the complement and hematopoietic systems. The capacity of cytokines to activate diverse cell types and to incite equally diverse responses underscores the pleiotropism of these inflammatory mediators. The bioactivities of different cytokines are significantly overlapping. According to their variations in effects *in vivo*, depending on time and location, there are basically three classes of cytokines: proinflammatory (T helper—Th1), anti-inflammatory (Th2), and Th17, different form both Th1 and Th2. Cytokines with proinflammatory effects are tumor necrosis factor- (TNF-) alpha, interleukin- (IL-) 1, IL-8, and HMGB-1; anti-inflammatory effects are present, for example, in IL-10 and IL-1 receptor antagonist (ra), and some cytokines possess both characteristics, like IL-6. The effects of cytokines are initiated by their binding to specific receptors at the membrane of target cell. This binding starts a cascade of events that leads to induction, enhancement, or inhibition of cytokine-regulated genes in the nucleus of the target cell, which results in modulation of cell immune activity [12–14].

Therefore, one of key events in bacterial sepsis is activation of immune cells, either by whole bacteria or with products derived from the bacteria, which both lead to local and systemic inflammation. Inflammatory response is not a uniform event: its characteristics differ from organ to organ, as well as from organ to peripheral blood. This finding leads to the concept of compartmentalization, with the most prominent difference between the blood compartment and tissues. During infection, immune cells interact not only with live and dead bacteria (killed by complement, defensins, antimicrobial peptides, or antibiotics) but also with bacterial products, either cell wall antigens of Gram-positive bacteria, such as peptidoglycans and lipoteichoic acid; endotoxin of Gram-negative bacteria or derived from inside cells like bacterial DNA and HSPs. Whole bacteria and PAMPs are potent activators of immune cells. They interact with specific sensors, like TLRs and nucleotide-binding oligomerization domain: NOD1 and NOD2 molecules, which induce the production of inflammatory cytokines. Final results are activation and overexpression of early response genes, which are mostly driven through the activation of nuclear factor *κ*B (NF-*κ*B). Anti-inflammatory mediators tend to predominate within the circulation in order to avoid initiation of new inflammatory foci, but their presence within tissues may not always be sufficient to prevent the onset of dangerous proinflammatory response in the different compartments. Contrary to normal conditions, in severe infection cytokines are produced in excess, so their presence in blood becomes detectable. However, the cytokines in circulation are merely the tip of the iceberg, and leukocyte-associated cytokines can be identified even when amounts in plasma are undetectable. Traditionally, sepsis has been represented by an excess production of proinflammatory mediators in blood, with the presence of cytokines within the blood compartment being a key factor in maintenance of proinflammatory process. But, the presence of circulating cytokines may also deactivate leukocytes from a further migration within tissues in response to local gradients of chemokines. Hence, both proinflammatory and anti-inflammatory responses are concomitantly present in sepsis [15].

Accurate cause of organ failure and death in majority of patients who died of sepsis remains unknown. The results of postmortem investigations have shown a relative paucity of cell death in most organs [16]. According to one theory, the organ dysfunction in sepsis may be the consequence of a so-called *cellular hibernation response *[17, 18]. In the most recent review, Hotchkiss with his coauthors delineated three potential inflammatory responses in sepsis [19]. Major factors determining the immune responses in sepsis include pathogen virulence, size of bacterial inoculums, and comorbidities. In the first scenario, the initial phase in previously healthy patients with severe sepsis is characterized by an excess hyperinflammatory—proinflammatory response with fever, hyperdynamic circulation, and shock. These features are present despite the fact that both proinflammatory and anti-inflammatory responses begin rapidly and concomitantly after sepsis onset. In this early phase of sepsis, patients die due to cardiovascular collapse, metabolic derangements, and multiple organ dysfunctions. Although no particular anti-inflammatory therapies have improved survival in large phase 3 trials, short acting anti-inflammatory or anticytokine therapies offer a theoretical benefit. Hotchkiss proposed a second scenario, in which many septic patients are elderly, with numerous comorbidities impairing their immune response. In these patients, sepsis development is commonly characterized by blunted or absent hyperinflammatory response, with predomination of anti-inflammatory phase. In this setting, boosting immunity with an immunoadjuvant therapy seems promising. Finally, the third theory of immune response in sepsis is featured by cycling between hyperinflammatory and hypoinflammatory states. In this scenario, septic patients first experience an initial hyperinflammatory response, followed by hypoinflammatory state. With the development of a new secondary infection, patients experience a new onset of hyperinflammatory reaction and may either recover or reenter the hypoinflammatory phase. Death may occur in either state. The longer the sepsis continues chances for a patient to develop profound immunosuppression increase. Autopsy results show that most patients admitted to intensive care units (ICUs) for treatment of sepsis had unresolved septic foci at postmortem. These findings suggest that septic patients were unable to eradicate invading pathogens and were more susceptible to nosocomial infections or both. In order to investigate modulation of the immunosuppressive phase of sepsis, Coopersmith and Hotchkiss with coworkers performed very interesting animal study using clinically relevant two-hit model of sepsis, that is, cecal ligation and puncture (CLP), followed by the induction of *Pseudomonas aeruginosa* pneumonia in mice. They applied an agent that blocks IL-10, a key mediator of immunosuppression, to investigate its ability to reverse immunoparalysis and improve survival. The improved survival was associated with restoration of interferon- (INF-) gamma synthesis, increased production of proinflammatory cytokines, and decreased bacterial growth. These authors found that immunosuppression, which occurs after the initial septic insult, increases susceptibility to secondary infection. However, seven days after CLP procedure, the host's immune system recovers sufficiently to generate an effective immune response. Modulation of the immunosuppressive phase of sepsis may help develop the therapeutic strategies [20].

Critically ill patients suffer a high rate of nosocomial infection. In fact, the common cause of death in these patients is secondary sepsis. This high prevalence of secondary infections argues for the influence of an immune suppression that may, at first glance, appear paradoxical in light of the proinflammatory nature of many critical illnesses. In ICU patients requiring organ support, the prevalence of nosocomial infections increases to 25–40% [21]. Research performed in the last 10 years revealed that many interventions applied in ICUs, such as high-volume crystalloid resuscitation, early total parenteral nutrition, liberal blood transfusions, high tidal volume mechanical ventilation, and intermittent hemodialysis, were, in fact, facilitating nosocomial infections and late MODS. There is growing evidence of the role of proinflammatory mediators in developing immune dysfunction. This observation may contribute to explanation of apparent paradox of immune suppression present in a patient with manifested hyper-inflammation [22]. Clinically, many patients show signs of persisting inflammation and immune-mediated organ damage while simultaneously remaining highly susceptible to secondary infections, suggesting the term *complex immune dysfunction syndrome (CIDS)* [23]. The novel investigations of sepsis point out that virtually all immune cells (both innate immune type such as neutrophils, monocytes, tissue macrophages, and dendritic cells and adaptive immune type like T cells, B cells, and natural killer (NK cells)) demonstrate immune hypoactivity. For example, neutrophils display dual state by concomitant presence of activation and dysfunction features. In critically ill patients, dysfunction of organs is, to a considerable degree, driven by neutrophils, which are key immune cells [24]. They tend to express surface markers of activation (increased levels of CD11b and CD64), but simultaneously they display major impairment of phagocytic capacity and generation of reactive oxygen species (ROS). This apparently paradoxical superposition of both proinflammatory activation and failure of key antimicrobial functions within the same cell type was illuminated by the finding that dysfunction was driven by an excess of the proinflammatory complement split product, anaphylatoxin, and C5a [25, 26]. Key role of the systemic complement activation in acute organ dysfunction during sepsis has been revealed 20 years ago [27].

Most recently, Lyle Moldawer and Frederick More, with their coworkers, proposed that *“persistent inflammation-immunosuppression catabolism syndrome*—*PICS”* is the predominant phenotype that has replaced late occurring MODS in surgical ICU patients who fail to recover [28]. Key effector cells that remove pathogens and present antigens in innate immunity are terminally differentiated macrophages (Kupffer cells and splenic macrophages), blood monocytes, and dendritic cells. Macrophage dysfunction is a significant contributor to both innate immunosuppression and adaptive immunosuppression. The state of immune paralysis is characterized by decreased bacterial clearance, decreased capacity to present antigens and to release proinflammatory cytokines. The main features of sepsis-induced immunosuppression are presence of defective T cells, with apoptotic depletion, decreased proliferation, and Th-2 polarization. Clinical relevance of PICS was elucidated by Moldawer and More. Over the years, the management of SIRS in ICUs has become more and more successful. That means the more patients reside in ICUs for weeks, with clinical manifestations of moderate SIRS and/or secondary infection, requiring life support. They commonly develop progressive protein catabolism resulting in substantial loss of lean body mass followed by additional weakening instead of regaining strength. Considering these facts, the main challenge for clinicians today is to manage simultaneous chronic inflammation and adaptive immunosuppression, as well as to provide the protection against secondary nosocomial infection and prevent severe protein catabolism.

Myeloid-derived suppressor cells (MDSCs) are other important regulators of the immune system, representing heterogeneous myeloid-originated population of cells that comprise myeloid progenitor cells, immature macrophages, immature granulocytes, and immature dendritic cells. When activated, they produce reactive oxygen and nitrogen species and arginase 1. They are also potent suppressors of various T-cell functions, predominantly antigen-specific CD8+ and CD4+ T-cell responses [29]. In his work, Moldawer, with his coworkers, was particularly interested in a paradoxical role of MDSCs in sepsis and trauma [30]. He reported that there is important role of MDSCs in inflammatory processes, both acute and chronic, and suggested that MDSC expansion is rather a programmed response to inflammation, regardless of its source, contrary to the previously established opinion that it is simply a pathologic response to a growing tumor. Mature myeloid cells are a relatively diverse population; half-life of blood neutrophils is few hours, while, in terminally differentiated macrophages and dendritic cells, half-life is up to months and even years. Nevertheless, during infection and inflammation, there is rapid increase in requirements for and the consumption of these cells, so the host responds to PAMPs, alarmins, and DAMPs with emergency increase in production of myeloid cells. This response is probably mediated by growth factor (e.g., granulocyte/granulocyte-macrophage colony stimulating factor (G/GM-CSF)) and cytokines (IL-6 and IL-17) produced during the early SIRS response. In emergency myelopoiesis, MDSCs are present in bone marrow, secondary lymphoid organs, and even organs of the reticuloendothelial system. Unlike terminally differentiated macrophages and monocytes, these cells produce large amounts of IL-10 and TNF-alpha after sepsis or trauma. They also consume large quantities of arginine, producing nitric oxide (NO), ROS, and peroxynitrites, acting both in proinflammatory and immunosuppressive manner. Patients with sepsis and burn injury, in which the expansion of the MDSC population is prevented, show decreased survival. MDSCs may be crucial for maintenance of innate immunity and inflammatory responses to secondary infection.

## 2. TNF-Alpha in Severe Acute Pancreatitis as a Clinical Example of Immune Response in Sepsis

As previously mentioned, sepsis is frequently characterized by elevated blood concentrations of both pro- and anti-inflammatory cytokines which may be associated with increased mortality. Cytokines activate multiple cellular processes and also activate other inflammatory mediators that contribute to organ dysfunction. Hence, patients with severe infection often develop MODS, which lead to further increase in morbidity and mortality. Actual underlying cause of severe sepsis may be different, for example, severe acute pancreatitis, secondary peritonitis, and trauma-induced sepsis, but they all may lead to systemic inflammation. Several clinical trials have been conducted in patients with severe sepsis, septic shock, and MODS in order to investigate the efficacy of biomodulators in blocking or inhibiting inflammation, but they all generally failed to improve the outcome. Recently, the trials have been performed to investigate the role of counterinflammatory signaling and newer concept of the cholinergic anti-inflammatory pathways [31].

TNF is one of the best described proinflammatory cytokines. It not only is a potent stimulator of the activation of many cell types such as macrophages/monocytes and NK cells but also can induce cell survival or cell death by apoptosis. This cytokine is tightly related to regulation of host innate immunity, inflammation, and apoptosis. It is primarily produced as a 212-amino acid type 2 trimeric transmembrane protein. The release of soluble form is enabled by proteolytic cleavage, mediated by the metalloprotease TNF converting enzyme (TACE, also called ADAM17) [32]. There are numerous different physiological stressors which may stimulate the secretion of TNF, such as endotoxin (LPS), hypoxemia, ischemia/reperfusion, hemorrhage, and complement system. Once secreted, TNF has multiple effects on the host response like increasing synthesis of a potent vasodilator NO, activating the arachidonic acid pathway, and inducing activation of cyclooxygenase and lipoxygenase. These processes increase the production of thromboxane A2 and prostaglandin E2 and augment their physiological effects. TNF also induces the production of selectins, platelet activating factor, and intracellular adhesion molecules (ICAM), which mediate neutrophil migration into tissues. This indicates that TNF plays a major role in activation of both thrombotic and fibrinolytic pathways on endothelial and epithelial cells. Besides macrophages and monocytes, there are other cells capable of TNF production, such as T cells after activation. TNF is early proximal cytokine with a short half-life (less than 20 minutes). This short half-life is long enough for induction of synthesis of the variety of pro- and anti-inflammatory cytokines like IL-6, IL-8, IFN-gamma, and IL-10. The other effects of TNF include altering the levels of corticosteroids. Not only these effects are the consequence of systemic release but also local release of TNF may lead to organ failure, independent of its blood concentrations. TNF acts via its receptors TNFR1 and TNFR2, which belong to a still growing number of TNF receptors in the TNFR superfamily. TNF recognition can lead to divergent results, depending on the specific receptor and environmental factors. TNFR1 has a death domain at the cytoplasmic tail. By binding to this receptor, TNF-alpha induces the transcription factors like NF-*κ*B and subsequent transcription of inflammatory genes, which seems to protect cell against programmed death. This binding can also induce the apoptosis by caspase cascade activation. Activation of TNFR1 only signals for cell death under distinct circumstances, for example, when the protein synthesis is blocked or when NF-*κ*B activation is inhibited. Contrary to TNFR1, which is expressed in most tissues and can be activated by both membrane-bound and soluble trimeric forms of TNF-alpha, TNFR2 is expressed in immune competent cells and on the endothelium and becomes activated by membrane-bound TNF-alpha. Also, this receptor does not contain a death domain. Binding to TNF initiates conformational changes in its receptors, thus inducing downstream signaling which activates at least three different pathways including NF-*κ*B, mitogen-activated protein kinases (MAPK), and death signaling [33].

Contact of TNF and TNFR2 activates signal transduction pathways such as NF-*κ*B and Jun N-terminal kinase and also induces activation and proliferation of immune cells (neutrophils, NK cells, B cells, and peripheral T cells). In patients with chronic inflammatory diseases, but not in patients with sepsis, there was a considerable success with the administration of anti-TNF antibodies or soluble TNF receptors in order to inhibit TNF activity.

Acute pancreatitis (AP) is a disease with incidence varying from 5 to over 100/100 000 people per year. Severity and mortality rates also vary. The most frequent is mild form of the disease, but severe acute pancreatitis (SAP), complicated with local lesions and/or organ failure, is developed in about 20% of the cases. Clinical course of SAP may be fulminant, and the releasing of inflammatory mediators into the bloodstream may affect distant organs. Hence, the major cause of death in these patients is MODS which occurs as a complication of SAP in 20–80% of the cases [34]. Recently, we performed a study regarding plasma levels of TN-alpha, as one of the most important cytokines in pathogenesis of acute pancreatitis, in patients with severe acute pancreatitis (SAP) on admission as predictors of severity and outcome of SAP. Blood samples were obtained from 100 patients with SAP. According to severity, patients were divided into two groups: 69 patients were in SAP group and 31 in SAP-induced MODS group. Fifty-three patients were alive 90 days after taking the blood sample for cytokine measurement and thus were recorded as survivors. When comparing SAP group with SAP-induced MODS group, we found that mean values of TNF-alpha on admission were 191.5-fold lower in group with SAP-induced MODS (*P* < 0.01). When comparing nonsurvivors with survivors, we found that mean values of TNF-alpha on admission were 63-fold higher in survivors (*P* < 0.01). At cut-off level of 7.95 pg/mL sensitivity was 83.9% and specificity was 72.5%. Patients with TNF-alpha level lower that 7.95 pg/mL had 3.2-fold higher probability to develop SAP with MODS. At cut-off level of 10.5 pg/mL, sensitivity was 83.0% and specificity was 77.4%. Patients with TNF-alpha level higher than 10.5 pg/mL had 4.8-fold higher probability to survive. We concluded that TNF-alpha is good predictor of severity and outcome. Low TNF-alpha concentration in patients with SAP predicts development of MODS and fatal outcome in our study [35]. Several studies have produced conflicting results regarding levels of cytokines in circulation and severity and outcome of systemic inflammation in critically ill patients. Contrary to some authors [36, 37] who found that high serum TNF-alpha levels correlate positively with the severity of disease and fatal outcome, we showed in our investigation that patient with SAP-induced MODS and fatal outcome had very low serum TNF-alpha levels. Florence Riche with coauthors found, as did we in our study, that in patients with abdominal septic shock high serum TNF levels were associated with increased survival [38]. The high serum level of TNF may reflect the efficacy of peritoneal inflammatory response against abdominal sepsis, and SAP belongs to that category. Ten years ago Dugernier and coauthors published the results of their interesting study regarding compartmentalization of the inflammatory response during acute pancreatitis. Their investigation was conducted in large cohort of 60 patients with SAP in whom they did peritoneal lavage and thoracic duct drainage at the onset of MODS [39]. In order to assess the pro- and anti-inflammatory responses, the site of mediator production, and their route of diffusion, they collected simultaneous samples of ascites, thoracic lymph, and blood at the onset of MODS and for the following 6 days. In less than 15% of blood and lymph samples they detected TNF-alpha and IL-1beta. Levels of secondary pro- and anti-inflammatory cytokines were elevated in all compartments from the beginning of investigation and throughout the entire sampling period. Cytokine concentrations were the highest in ascites and decreased from lymph to blood, suggesting a splanchnic origin. Although a net proinflammatory activity ascribed to IL-1beta was detected in ascites, a net anti-inflammatory activity was measured virtually in all lymph and blood samples. That indicates that the pancreas and the splanchnic area are sites of proinflammatory response while an early and sustained anti-inflammatory activity dominates in circulating compartments. This suggests that local proinflammatory stimuli induce rapid, robust, and dominant anti-inflammatory response in circulatory compartments, which may lead to an increased risk of developing secondary infection [40]. The timing of measurement of the proinflammatory cytokines from the onset of the disease is of great importance. In SAP, TNF-alpha is released in circulation in the first few hours and rapidly disappears after that. Because our patients presented to the ED at various stages of SAP, in some cases the first samples might be collected after this early TNF-alpha peak. In addition, the presence of soluble TNF receptors can interfere with the detection of unbound TNF. The production of cytokines at various tissue sites depends, in part, on the proximity of given site to the injurious stimulus. The magnitude of injury may also influence the increase in cytokine levels. However, it has been difficult to correlate plasma concentration of a particular proinflammatory cytokine with the overall extent of tissue damage in clinical trials. This is supported by the fact that cytokines are a component of a paracrine system that is involved in signaling the local presence of inflammation to adjacent somatic tissue. Also, cytokines may occur both as free secreted and cell-associate forms. TNF-alpha exists as a high-molecular weight, cell-associated membrane form in inflammatory cells. This form of TNF-alpha acts by direct cell-to-cell contact. Its dual nature also helps explain why systemic concentration of circulating TNF-alpha may not be reflective of the degree of local TNF-alpha activity.

## 3. Immune Response in Severe Trauma with or without Secondary Sepsis

In 1536 the army of King Francis I of France fought at the city of Turin against the army of the Holy Roman Emperor Charles V. After the French army recaptured the city in 1537, their surgeon general, Amroise Pare, wrote a passage in which he reported inevitable consequence of nonfatal wound, dreaded by soldiers. We now entitle Amroise Pare to be the father of modern trauma surgery, and the described entity is now termed *posttraumatic sepsis* [41].

Worldwide, in the general population under the age of 45, trauma is one of the main causes of death. Mortality rate after major trauma is different regarding time period after injury. First, we recognize the immediate effects of trauma with death at the scene or within the first hour with mortality rate of 53–72%. These deaths are common consequences of massive head injury or bleeding. The second peak is somewhat smaller and occurs in the first 24 hours. The deaths are mainly due to hypoxia, hypovolemia, or traumatic brain injury. In survivors, we recognize the third pattern, characterized by high risk of developing immune dysfunction and subsequently sepsis, leading to MODS with high mortality rate. All severe posttraumatic complications (systemic inflammatory response syndrome (SIRS), acute respiratory distress syndrome (ARDS), and MODS) are directly related to synthesis and release of inflammatory mediators into the bloodstream, which is in fact the *first hit*—representing the severity of injury itself, and then there is *second hit*, such as surgical intervention and/or bacterial infection [42–44]. Based on this concept, a new immunoinflammatory paradigm in critically injured patients is developed [45]. Current concept explains the complications of severe injury as the consequence of excessive proinflammatory response (SIRS), representing excessive innate immune response, then followed by compensatory anti-inflammatory response syndrome (CARS), representing suppressive adaptive immune response. A second-hit phenomenon results from sequential insults that lead to more severe, recurrent SIRS and organ dysfunction. The proposed new paradigm considers rapid and simultaneous induction of innate genes (both pro- and anti-inflammatory) and suppression of adaptive immunity genes. Recoveries from complications are delayed, and patients are captured in prolonged state of immune-inflammatory dysregulation. Patient is defending against the bacterial invasion by his/her first line of defense, epithelial barrier, but it is often disrupted in trauma, allowing the penetration of microorganisms. Other lines of defense include activation of immune cells and production of cytokines.

Six years ago we focused our research on immune cytokine response in very specific group of injured patients, namely, combat casualties, regarding secondary sepsis development [46]. Combat operations are becoming more frequent worldwide. Considering this, we wanted to evaluate the immune response in combat casualties who suffered from blast or explosive trauma, with or without secondary sepsis, and to assess the prognostic values of certain proinflammatory (TNF-alpha and IL-8) and anti-inflammatory (IL-4 and IL-10) cytokines, regarding severity and outcome. To our knowledge, until that time, next to nothing was done in research of cytokine response to combat trauma with or without sepsis in war time condition.

The study group consisted of 76 male combat casualties. The moment of sustaining injury was establish in 76% of the cases. In 61% of the cases time interval between sustaining injury and admission, when the first set of blood samples was taken, was 6 hours. In these patients, initial surgical treatment was administered in Military Medical Academy (MMA) in Belgrade. In the rest of the cases, time interval between injury and taking the first blood sample was 12 hours, for these patients were initially surgically treated in front line hospitals and then transferred to MMA. Group I consisted of 56 casualties with blast of explosive trauma who developed secondary sepsis (trauma + sepsis group). The criteria for establishing diagnosis of sepsis included positive blood culture. Group II consisted of 20 casualties, selected to match Group I. They all suffered from blast or explosive trauma equally severe as in Group I, but without sepsis (trauma group). Trauma severity was determined according to injury severity score (ISS). In Group I there were 15 and in Group II 5 true blast victims (without any evidence of being struck by any object) in which ISS was determined intraoperatively. There was no statistically significant difference in ISS between two groups (mean ± SD): 29 ± 10.4 in Group I (trauma + sepsis) and 31.7 ± 12.5 in Group II (trauma), respectively. Also, there was no statistically significant difference in percentage of abdominal wounds between two groups (35.7% in Group I versus 40.0% in Group II). Severity of shock according to SOFA score was significantly higher in Group I (trauma + sepsis): 6.09 ± 3.73 versus 2.95 ± 3.87 (mean ± SD), *P* < 0.01.

According to severity of clinical status the patients were also divided into two groups, SIRS (less severe) and MODS (more severe) group. When compared trauma + sepsis group with trauma group, we found statistically highly significant difference (*P* < 0.01) in IL-8, IL-10 and statistically significant difference (*P* < 0.05) in TNF-alpha concentrations; mean values of IL-8 were 230-fold, IL-10 42-fold, and TNF-alpha 17-fold higher in trauma + sepsis group. When comparing MODS with SIRS group, we found statistically highly significant difference (*P* < 0.01) in IL-8, TNF-alpha, and IL-10 concentrations; mean values of IL-8 were 60-fold, TNF-alpha 43.5-fold, and IL-10 70-fold higher in MODS group. Concentrations of the same three cytokines were significantly different (*P* < 0.01) when we compared nonsurvivors with survivors; mean values of IL-8 were 2.3-fold and IL-10 1.4-fold higher in non-survivors, while mean values of TNF-alpha were 2.2-fold higher in survivors. Unexpectedly, concentrations of IL-4 were not statistically different between groups regarding secondary sepsis, severity, and outcome.

Meduri proposed interesting explanation for poor outcome in our patients with excessive proinflammatory and anti-inflammatory response. Since some studies have shown positive correlation between sustained and intense inflammatory responses and the incidence of bacterial infections, he hypothesized that cytokines secreted by the host during MODS may indeed favor the growth of bacteria and hence establish a relationship between exaggerated and protracted systemic inflammation and the frequent development of infections [47].

Last year, Charles Wade and his group published comprehensive, systematic review of the literature regarding comparison of mortality associated with sepsis in the burn, trauma, and general intensive care unit patients [48]. Sepsis outcomes in these three distinct patient populations were compared for the first time. Conclusion of this review was that trauma patients showed relatively low mortality associated with sepsis, while in burn patients as well as in the older critical care patients the prevalence of sepsis was higher with worse outcomes. Out of 334 titles, 97 abstracts, and 65 full texts retrieved, 38 studies fulfilled strict criteria to be reviewed. Our study was included with the highest level of evidence rating (IV: evidence from well-designed studies) and quality (A grade). The same group of authors suggested that combining markers of inflammation and coagulation and standard clinical indices would improve early prediction of in-hospital mortality in burn and nonburn trauma (but not combat trauma) patients [49]. They concluded that, compared to previous methods, the proposed model improves prediction of in-hospital mortality. In spite of our best efforts, we could not find, in the literature available to us, studies regarding systemic effects of cytokines in combat casualties. What we did find were several articles regarding inflammatory biomarkers and combat wound dehiscence and healing. Most of them concluded that the cytokine and chemokine protein and gene transcript expression patterns demonstrate a condition of inflammatory dysregulation associated with war wound failure and that molecular biomarker panel may predict wound healing outcome and warrants prospective validation [50–53]. In one study, encouraged by the correlation between systemic and local inflammatory cytokines and microbial colonization assessed by quantitative cultures, authors proposed the concept of interplay between the systemic response to injury and local wound environment as a determinant of outcome [54]. The same authors also stated that this relationship remains poorly defined and requires further investigation in both clinical and preclinical studies; we completely agree with that statement.

Severe trauma, commonly followed by substantial blood loss, leads to decreased endothelially derived NO, which further leads to increased platelet aggregation, increased neutrophil infiltration, and deregulation of vasorelaxation. As a result, the increase of microvascular permeability, concomitantly with ultimate loss of endothelial integrity, simultaneously occurs. The first line of defense against invading microorganism is formed by the innate immune responses, which rapidly react to DAMPs. Adaptive immunity responds slower because antigen-specific reaction requires initial sensibilisation [55]. Antigen presentation after injury is the function of monocytes and macrophages as their mature phagocytotic phenotype. They recognize, uptake, and kill invading microorganisms, which initiate an adequate immune response. In cases when this monocyte function is impaired, antigen-presenting ability is decreased, together with disrupted monocyte—T cell interaction; that has been related to development of septic complications after severe trauma. Restitution of monocyte function is reflected by the significant increase of TNF-alpha, for example, after a temporary state of predominant anti-inflammatory production of IL-4 and IL-10 by Th2 cells. Endogenous inflammatory mediators such as TNF-alpha and NO activate premature apoptosis of immune effector cells, which may contribute to the sepsis-associated MODS after severe trauma. Neurohumoral signaling, via binding of glucocorticoids, catecholamines, or adrenergic agonists to the corresponding receptors on immune cells, can, for example, suppress cytokine production and thus impair a competent immune regulatory cell-cell interaction [56]. The concept of T-cell mediated immunosuppression is now somewhat improved by reports of the activity of Th17 cells. This lineage of inflammatory CD4+ T-cell subpopulation exhibits particular developmental and phenotypic characteristics different from both Th1 and Th2-types and is capable of IL-17 production [57]. Numerous authors use cytokines as prognostic markers regarding outcome of trauma in patients with SIRS, sepsis, or MODS. Some authors favor IL-6 in this regard and propose threshold level of 800 pg/mL on admission to be a good indicator for differentiating between patients with or without organ failure [58].

Besides its role in sepsis (as we elucidated in the first part of this review), complement also plays a significant role in activation of the innate immune system in contributing to the pathogenesis of trauma-induced sequelae and adverse outcome. Complement system takes part in the first line of defense, where it acts as a potent effector of innate immunity, which implicates this system in mediation of the early posttraumatic inflammatory response. Despite its generic beneficial functions, including pathogen elimination and immediate response to danger signals, complement activation may exert detrimental effects after trauma in terms of mounting an “innocent bystander” attack on host tissue. Ischemia-reperfusion injury after trauma is classical example of tissue damage mediated by complement activity. Complement activity may also exacerbate local and systemic inflammation and release of toxic mediators, thus adding to the “antigenic load.” This activity may consequently sustain SIRS after major trauma, ultimately contributing to remote organ injury and death. This pathophysiological pattern represents the fundament of new therapeutic approach named *site-targeted complement inhibition* [59].

Severe trauma commonly leads to major impairment of the immune system. Hyperinflammation state after trauma mediates remote organ damage and may lead to MODS, while, on the other hand, immunosuppression enhances risk of developing acquired infectious complications. Pathophysiological substrates for these opposite consequences of trauma involve the role of endogenous danger signals, such as HMGB-1 and HSPs, generated in destroyed tissues, which mediate trauma-induced immune dysfunction [60]. The major danger signals that initiate immune response after trauma are dual-function alarmins HMGB-1, IL-1 alpha, and IL-33. They play the most important role in activation and propagation of the inflammatory response after disruption of cellular integrity. The common characteristics of these three alarmins are their activity as transcription factors and extracellular mediators of inflammation; however, each dual-function protein exerts distinct functions. In addition, a new field for investigation of danger sensing and transmission is opened by the discovery of mitochondrial DAMPs, which activate immune response after cellular disruption by mimicking bacterial infection [61]. Mitochondria emerged as crucial mediators in the induction of apoptosis during traumatic shock [62]. Besides apoptosis, there is now evidence of presence of *necroptosis*, a form of organized cell necrosis. Necroptosis can be induced by TNFR and other so-called death receptors [63, 64]. The important role of mitochondria in activation of innate immunity is supported by the fact that they contain constituents of their bacterial ancestors which are potentially immunogenic [65]. Key receptor for danger signals is TLR-4. This receptor has been extensively studied, and ten TLR homologues have been identified in humans. In addition to its ability to recognize the bacterial LPS, it has been now revealed that TLR-4 can be activated by danger signal molecules released after cellular injury. Hemorrhagic shock and consequent resuscitation, that make common chain of events following severe trauma, may lead to global ischemia/reperfusion injury and MODS as a final result. The potential role of TLR-4 in this process is supported by its expression in liver, lungs, and myocardium during hemorrhagic shock and resuscitation [66]. The immune response may be influenced by the type of trauma. In the study of Mace and coauthors, burns were associated with a greater and more sustained immune-inflammatory response than nonburn trauma (evidenced by increased concentrations of IL-6 and IL-8 in plasma during the first week after trauma). They found no association between MODS and plasma cytokine concentrations [67].

There are several factors that contribute to the immune response and end organ damage after trauma. Some etiological factors are intrinsic, including genetic physiological status and predisposition, while others are extrinsic, such as type of injury (“trauma load” or “intervention load,” meaning surgery). The only factor that can be altered by the attending physician is the intervention load. The damage caused by immune response to trauma hence may be attenuated by the adjustment of the therapeutic approach and surgical treatment strategy [68]. 

## 4. Is Immune Response in Sepsis and Trauma, at Least in Part, Genetically Determined?

The inflammatory response contributes significantly to the morbidity and mortality of critically ill patients and may extremely vary between individuals. In patients with similar infection, there is tremendous variability reported in the clinical profile and outcome. The risk of sepsis and its outcome are influenced by host predisposition [69, 70]. That predisposition may be explained by interindividual genetic variability, represented by genetic polymorphisms [71]. Genetic polymorphisms in the immune response to infection are associated with the susceptibility to certain infection and with clinical outcome. Understanding the biology of inflammation is significantly improved, but so far it is not followed by substantial improvements in clinical outcome; furthermore, the sporadic promising results have been related to supportive care efforts rather than to specific therapies. As a result, mortality and cost of treatment of patients suffering from severe infections remain high.

Twenty-five years ago, Sorensen and coauthors reported that in adult adoptees the risk of dying from infection has been 5.81-fold higher when one of their biologic parents died of infection before the age of 50. This risk exceeded the relative risk (RR) of dying of malignancy (1.19) and cerebral and/or cardiovascular causes (4.5). These findings suggest a significant genetic susceptibility to lethal infection and sepsis [72]. Studies performed in order to determine polymorphisms related to sepsis have been mainly focused on one or more polymorphisms for specific genes that generate proteins involved in immune response in sepsis such as pro- and anti-inflammatory cytokines and elements of innate immunity and coagulation/fibrinolysis pathways. Severe injury also activates the innate immune response as part of the inflammatory response, which in turn can lead to secondary MODS, so there may be genetic predisposition to adverse effects due to trauma [73]. The connection between phenotype and sepsis has been established by genetic mapping of the single nucleotide polymorphisms of IL-6, IL-18, TNF-alpha, IFN-gamma, and TLRs [74].

The most common form of stable genetic variation in the population is a *single nucleotide polymorphism* (SNP), which refers to single-base-pair positions in genomic DNA in which sequence alternatives exist with a frequency of more than 1%. SNPs are not the cause of disease itself, but they may alter the risk for disease development. They may also influence the outcome of a disease. Of all SNPs in the human genome, it has been estimated that 10% have the potential of modifying some biologic processes [75].

Three years ago we investigated whether distributions of TNF-alpha_308_, IL-10_1082_, CD14_159_, and IL-1ra gene intron 2 genotypes in critically ill are associated with outcome, underlying cause of sepsis, type of microorganism. Blood samples from 106 critically ill Caucasian patients (severe acute pancreatitis, secondary peritonitis, and trauma with or without sepsis) were genotyped by methodology based on polymerase chain reaction (PCR) for TNF-alpha_308_, IL-10_1082_, cluster of differentiation, CD14_159_, and IL-1 receptor antagonist gene intron 2. All patients with TNF-alpha_308_AA genotype survived; RR of death in patients with AG was 3.250 and with GG 1.923 (*P* < 0.01). In patients with Gram-positive sepsis IL-10_1082_AA and then AG genotypes were the most frequent ones (OR 18.67 and 7.20, resp., *P* < 0.01). When comparing IL-10_1082_AA with AG, RR of pancreatitis being underlying cause of sepsis was 1.80; OR was 3.40. When AA and GG were compared, RR was 7.33; OR was 20.00. In patients with GG, RR of peritonitis being underlying was 4.07; OR was 5.88 (*P* < 0.01). In patients with Gram-positive sepsis CD14_159_CT was the most frequent one with OR 5.25. Distribution of six IL-1ra gene intron 2 genotypes showed no significant association. We concluded that distribution of TNF-alpha_308_ genotypes is associated with outcome, IL-10_1082_ with type of microorganism and underlying cause of sepsis, and CD14_159_ with type of microorganism [76]. We are aware that there are inconsistent findings in current studies of genetic association in human trauma and/or sepsis (same as ours, opposite to ours, or with no association at all). Strict critics have focused on methodological and analytical problems (namely, underpowered studies), but they also state that, for example, it has been calculated, for a general ICU population with sepsis or septic shock, that a sample size of 2000 patients would be required to detect a mortality RR of 1.5 from any polymorphism to confidently exclude false negative associations. To our knowledge no genetic association study recruited this number of patients. Until then, relatively small population studies should be taken into account. 

Identification of strong associations between certain genetic polymorphisms and increased mortality rate, underlying cause of sepsis or the type of infecting microorganism, is intriguing and requires further research. Despite previously mentioned limitations of the most studies of association between genetic polymorphisms and sepsis, this approach is promising. Genetic and molecular aspects of host immunoinflammatory response to various insults in critically ill patients are complex, and establishing a certain association does not mean revealing the causative relationship. Genetic studies might allow for earlier differentiation among patients with immunoinflammatory response to either infection or trauma, allowing for more focused and timely treatment. Molecular profiles might be established to distinguish a good versus a poor response to therapeutic intervention.

## References

[1] Geroulanos S, Douka ET (2006). Historical perspective of the word ‘sepsis’. *Intensive Care Medicine*.

[2] Cohen S (2004). Cytokine: more than a new word, a new concept proposed by Stanley Cohen thirty years ago. *Cytokines*.

[3] Carswell EA, Old LJ, Kassel RL (1975). An endotoxin induced serum factor that cuases necrosis of tumors. *Proceedings of the National Academy of Sciences of the United States of America*.

[4] Funk DJ, Parrillo JE, Kumar A (2009). Sepsis and septic shock: a history. *Critical Care Clinics*.

[5] Nduka OO, Parrillo JE (2009). The pathophysiology of septic shock. *Critical Care Clinics*.

[6] Ulloa L, Tracey KJ (2005). The “cytokine profile”: a code for sepsis. *Trends in Molecular Medicine*.

[7] Hoesel LM, Ward PA (2004). Mechanisms of inflammatory response syndrome in sepsis. *Drug Discovery Today*.

[8] Opal SM (2007). The host response to endotoxin, antilipopolysaccharide strategies, and the management of severe sepsis. *International Journal of Medical Microbiology*.

[9] Klune JR, Dhupar R, Cardinal J, Billiar TR, Tsung A (2008). HMGB1: endogenous danger signaling. *Molecular Medicine*.

[10] Matzinger P (2002). The danger model: a renewed sense of self. *Science*.

[11] Surbatovic M, Radakovic S, Jovanovic K (2005). New strategies in multiple organ dysfunction syndrome therapy for sepsis. *Srpski Arhiv za Celokupno Lekarstvo*.

[12] Surbatovic M, Filipovic N, Slavkovic Z (2006). Infection and inflammation in sepsis. *Vojnosanitetski Pregled*.

[13] Djordjevic D, Surbatovic M, Ugrinovic D (2012). New aspects of sepsis pathophysiology in critically ill. *Vojnosanitetski Pregled*.

[14] Ulloa L, Cai B, Deitch EA (2010). Novel insights for systemic inflammation in sepsis and hemorrhage. *Mediators of Inflammation*.

[15] Cavaillon J-M, Annane D (2006). Compartmentalization of the inflammatory response in sepsis and SIRS. *Journal of Endotoxin Research*.

[16] Hotchkiss RS, Swanson PE, Freeman BD (1999). Apoptotic cell death in patients with sepsis, shock, and multiple organ dysfunction. *Critical Care Medicine*.

[17] Fink MP, Evans TW (2002). Mechanisms of organ dysfunction in critical illness: report from a Round table Conference held in Brussels. *Intensive Care Medicine*.

[18] Abraham E, Singer M (2007). Mechanisms of sepsis-induced organ dysfunction. *Critical Care Medicine*.

[19] Hotchkiss RS, Monneret G, Payen D (2013). Immunosuppression in sepsis: a novel understanding of the disorder and a new therapeutic approach. *The Lancet Infectious Diseases*.

[20] Muenzer JT, Davis CG, Chang K (2010). Characterization and modulation of the immunosuppressive phase of sepsis. *Infection and Immunity*.

[21] Vincent J-L, Rello J, Marshall J (2009). International study of the prevalence and outcomes of infection in intensive care units. *Journal of the American Medical Association*.

[22] Ward PA (2011). Immunosuppression in sepsis. *Journal of the American Medical Association*.

[23] Morris AC, Simpson AJ, Walsh TS, Vincent JL (2013). Hyperinflammation and mediators of immune suppression in critical illness. *Annual Update in Intensive Care and Emergency Medicine*.

[24] Brown K, Brain S, Pearson J, Edgeworth J, Lewis S, Treacher D (2006). Neutrophils in development of multiple organ failure in sepsis. *The Lancet*.

[25] Conway Morris A, Kefala K, Wilkinson TS (2009). C5a mediates peripheral blood neutrophil dysfunction in critically ill patients. *American Journal of Respiratory and Critical Care Medicine*.

[26] Morris AC, Brittan M, Wilkinson TS (2011). C5a-mediated neutrophil dysfunction is RhoA-dependent and predicts infection in critically ill patients. *Blood*.

[27] Lohde E, Raude H, Luck M (1992). Complement activated granulocytes can cause autologous tissue destruction in man. *Mediators of Inflammation*.

[28] Gentile LF, Cuenca AG, Efron PA (2012). Persistent inflammation and immunosuppression: a common syndrome and new horizon for surgical intensive care. *Journal of Trauma and Acute Care Surgery*.

[29] Gabrilovich DI, Nagaraj S (2009). Myeloid-derived suppressor cells as regulators of the immune system. *Nature Reviews Immunology*.

[30] Cuenca AG, Delano MJ, Kelly-Scumpia KM (2011). A paradoxical role for myeloid-derived suppressor cells in sepsis and trauma. *Molecular Medicine*.

[31] Fry DE (2012). Sepsis, systemic inflammatory response, and multiple organ dysfunction: the mystery continues. *American Surgeon*.

[32] Qiu P, Cui X, Barochia A, Li Y, Natanson C, Eichacker PQ (2011). The evolving experience with therapeutic TNF inhibition in sepsis: considering the potential influence of risk of death. *Expert Opinion on Investigational Drugs*.

[33] Parameswaran N, Patial S (2010). Tumor necrosis factor-a signaling in macrophages. *Critical Reviews in Eukaryotic Gene Expression*.

[34] Kylänpää M-L, Repo H, Puolakkainen PA (2010). Inflammation and immunosuppression in severe acute pancreatitis. *World Journal of Gastroenterology*.

[35] Surbatovic M, Radakovic S (2013). Tumor necrosis factor-alpha levels early in severe acute pancreatitis: is there predictive value regarding severity and outcome?. *Journal of Clinical Gastroenterology*.

[36] Terregino CA, Lopez BL, Karras DJ, Killian AJ, Arnold GK (2000). Endogenous mediators in emergency department patients with presumed sepsis: are levels associated with progression to severe sepsis and death?. *Annals of Emergency Medicine*.

[37] Shen Y, Cui N, Miao B, Zhao E (2011). Immune dysregulation in patients with severe acute pancreatitis. *Inflammation*.

[38] Riche F, Panis Y, Laisne M-J (1996). High tumor necrosis factor serum level is associated with increased survival in patients with abdominal septic shock: a prospective study in 59 patients. *Surgery*.

[39] Dugernier TL, Laterre P-F, Wittebole X (2003). Compartmentalization of the inflammatory response during acute pancreatitis: correlation with local and systemic complications. *American Journal of Respiratory and Critical Care Medicine*.

[40] Munford RS, Pugin J (2001). Normal responses to injury prevent systemic inflammation and can be immunosuppressive. *American Journal of Respiratory and Critical Care Medicine*.

[41] Thornhill R, Strong D, Vasanth S, MacKenzie I (2010). Trauma sepsis. *Trauma*.

[42] Giannoudis PV, Tosounidis TI, Kanakaris NK, Kontakis G (2007). Quantification and characterisation of endothelial injury after trauma. *Injury*.

[43] Giannoudis PV, Pape HC (2007). Trauma and immune reactivity: too much, or too little immune response?. *Injury*.

[44] Lenz A, Franklin GA, Cheadle WG (2007). Systemic inflammation after trauma. *Injury*.

[45] Xiao W, Mindrinos MN, Seok J (2011). A genomic storm in critically injured humans. *Journal of Experimental Medicine*.

[46] Surbatovic M, Filipovic N, Army S, Radakovic S, Stankovic N, Slavkovic Z (2007). Immune cytokine response in combat casualties: blast or explosive trauma with or without secondary sepsis. *Military Medicine*.

[47] Meduri GU (2002). Clinical review: a paradigm shift: the bidirectional effect of inflammation on bacterial growth. Clinical implications for patients with acute respiratory distress syndrome. *Critical Care*.

[48] Mann EA, Baun MM, Meininger JC, Wade CE (2012). Comparison of mortality associated with sepsis in the Burn, trauma, and general intensive care unit patient: a systematic review of the literature. *Shock*.

[49] Park MS, Salinas J, Wade CE (2008). Combining early coagulation and inflammatory status improves prediction of mortality in burned and nonburned trauma patients. *The Journal of Trauma*.

[50] Forsberg JA, Elster EA, Andersen RC (2008). Correlation of procalcitonin and cytokine expression with dehiscence of wartime extremity wounds. *Journal of Bone and Joint Surgery A*.

[51] Hawksworth JS, Stojadinovic A, Gage FA (2009). Inflammatory biomarkers in combat wound healing. *Annals of Surgery*.

[52] Hahm G, Glaser JJ, Elster EA (2011). Biomarkers to predict wound healing: the future of complex war wound management. *Plastic and Reconstructive Surgery*.

[53] Evans KN, Forsberg JA, Potter BK (2012). Inflammatory cytokine and chemokine expression is associated with heterotopic ossification in high-energy penetrating war injuries. *Journal of Orthopaedic Trauma*.

[54] Brown TS, Hawksworth JS, Sheppard FR (2011). Inflammatory response is associated with critical colonization in combat wounds. *Surgical Infections*.

[55] Pillay J, Hietbrink F, Koenderman L, Leenen LPH (2007). The systemic inflammatory response induced by trauma is reflected by multiple phenotypes of blood neutrophils. *Injury*.

[56] Tschoeke SK, Ertel W (2007). Immunoparalysis after multiple trauma. *Injury*.

[57] Nakae S, Iwakura Y, Suto H, Galli SJ (2007). Phenotypic differences between Th1 and Th17 cells and negative regulation of Th1 cell differentiation by IL-17. *Journal of Leukocyte Biology*.

[58] Pape H-C, Tsukamoto T, Kobbe P, Tarkin I, Katsoulis S, Peitzman A (2007). Assessment of the clinical course with inflammatory parameters. *Injury*.

[59] Neher MD, Weckbach S, Flierl MA, Huber-Lang MS, Stahel PF (2011). Molecular mechanisms of inflammation and tissue injury after major trauma-is complement the “bad guy”?. *Journal of Biomedical Science*.

[60] Flohé SB, Flohé S, Schade UF (2008). Invitedreview: deterioration of the immune system after trauma: signals and cellular mechanisms. *Innate Immunity*.

[61] Hirsiger S, Simmen HP, Werner CML (2012). Danger signals activating the immune response after trauma. *Mediators of Inflammation*.

[62] Hubbard WJ, Bland KI, Chaudry IH (2004). The role of the mitochondrion in trauma and shock. *Shock*.

[63] Duprez L, Takahashi N, Van Hauwermeiren F (2011). RIP kinase-dependent necrosis drives lethal systemic inflammatory response syndrome. *Immunity*.

[64] Vandenabeele P, Galluzzi L, Vanden Berghe T, Kroemer G (2010). Molecular mechanisms of necroptosis: an ordered cellular explosion. *Nature Reviews Molecular Cell Biology*.

[65] Bonawitz ND, Clayton DA, Shadel GS (2006). Initiation and beyond: multiple functions of the human mitochondrial transcription machinery. *Molecular Cell*.

[66] McGhan LJ, Jaroszewski DE (2012). The role of toll-like receptor-4 in the development of multi-organ failure following traumatic haemorrhagic shock and resuscitation. *Injury*.

[67] Mace JE, Park MS, Mora AG (2012). Differential expression of the immunoinflammatory response in trauma patients: burn vs. non-burn. *Burns*.

[68] Hietbrink F, Koenderman L, Rijkers GT, Leenen LPH (2006). Trauma: the role of the innate immune system. *World Journal of Emergency Surgery*.

[69] Sutherland AM, Walley KR, Russell JA (2005). Polymorphisms in CD14, mannose-binding lectin, and Toll-like receptor-2 are associated with increased prevalence of infection in critically ill adults. *Critical Care Medicine*.

[70] Henckaerts L, Nielsen KR, Steffensen R (2009). Polymorphisms in innate immunity genes predispose to bacteremia and death in the medical intensive care unit. *Critical Care Medicine*.

[71] Namath A, Patterson AJ (2009). Genetic polymorphisms in sepsis. *Critical Care Clinics*.

[72] Sorensen TIA, Nielsen GG, Andersen PK, Teasdale TW (1988). Genetic and environmental influences on premature death in adult adoptees. *The New England Journal of Medicine*.

[73] Lui DF, Baker JF, Pereira A (2012). Multiorgan failure in trauma: from conception to genomic era. *Current Orthopaedic Practice*.

[74] Giannoudis PV, Van Griensven M, Tsiridis E, Pape HC (2007). The genetic predisposition to adverse outcome after trauma. *Journal of Bone and Joint Surgery B*.

[75] Hildebrand F, Mommsen P, Frink M, Van Griensven M, Krettek C (2011). Genetic predisposition for development of complications in multiple trauma patients. *Shock*.

[76] Surbatovic M, Grujic K, Cikota B (2010). Polymorphisms of genes encoding tumor necrosis factor-alpha, interleukin-10, cluster of differentiation-14 and interleukin-1ra in critically ill patients. *Journal of Critical Care*.

